# The Emotional Toll of Hell: Cross-National and Experimental Evidence for the Negative Well-Being Effects of Hell Beliefs

**DOI:** 10.1371/journal.pone.0085251

**Published:** 2014-01-22

**Authors:** Azim F. Shariff, Lara B. Aknin

**Affiliations:** 1 Department of Psychology, University of Oregon, Eugene, Oregon, United States of America; 2 Department of Psychology, Simon Fraser University, Burnaby, British Columbia, Canada; Middlesex University London, United Kingdom

## Abstract

Though beliefs in Heaven and Hell are related, they are associated with different personality characteristics and social phenomena. Here we present three studies measuring Heaven and Hell beliefs' associations with and impact on subjective well-being. We find that a belief in Heaven is consistently associated with greater happiness and life satisfaction while a belief in Hell is associated with lower happiness and life satisfaction at the national (Study 1) and individual (Study 2) level. An experimental priming study (Study 3) suggests that these differences are mainly driven by the negative emotional impact of Hell beliefs. Possible cultural evolutionary explanations for the persistence of such a distressing religious concept are discussed.

## Introduction

Though the psychology of religion has tended to treat religion as a single construct, evolutionary theories of religion have argued that religion is instead a multifaceted family category – comprised of different beliefs, teachings and rituals that have emerged for different reasons at different times, to serve different cultural purposes. ‘Religion’ is many things. Supporting this argument, new evidence demonstrates that these different aspects of religions have systematically distinct psychological effects. For example, recent research has explored the divergent impact of benevolent aspects of religion, such as beliefs in Heaven and comforting, forgiving gods, versus more malevolent religious beliefs, such as those in Hell and punitive supernatural agents [1]–[2].

Compared to the benevolent aspects, supernatural malevolence has been found to be associated with stronger rule-following and group coordination at the national level. For instance, in developing countries (where secular institutions tend to be weaker), a higher proportion of citizens who believe in Hell is associated with higher GDP growth [3]. Similarly, controlling for the belief in heaven as well as obvious third variables such as wealth and wealth inequality, a higher rate of belief in hell is associated with lower national crime rates [2]. These studies suggest that belief in supernatural punishment may curb unethical behavior, allowing for greater social stability and economic success.

However, belief in supernatural malevolence may not be without its costs. Research has shown that people with more malevolent views of God tend to report lower self-esteem, psychological coping and health resiliency [4]–[5]. Thus, beliefs in religious malevolence may have emotional costs, even as they have norm-following benefits.

Here we present three studies testing the divergent emotional correlates and consequences of Heaven and Hell beliefs. Specifically, we examine whether these beliefs differentially affect subjective well-being. Although religiosity is consistently tied to greater well-being [6]–[7], little research has examined which elements of religious belief offer mood benefits, which do not, and which may in fact be detrimental. In Study 1, we used a similar method as Shariff & Rhemtulla [2] to measure the relationship between Heaven and Hell belief and subjective well-being at the cross-national level. In Study 2, we used data from the World Values Survey [8] to test these relationships at the individual level. In Study 3, we used an experimental priming method to test the causal relationships between Heaven and Hell beliefs and subjective well-being.

We note that this exploration diverges from the growing literature examining the relationship between religion and well-being. Over the past several years, scholars from various disciplines, such as sociology [7], psychology [9], and economics [10], have explored the relationship between religious beliefs and happiness. Results typically reveal that religious beliefs are associated with greater well-being [6], [11]–[14]. Although this existing work makes great strides in assessing the impact of widespread religious beliefs with large-scale data sets, the present paper offers two important theoretical extensions. First, the present work examines the well-being consequences of specific religious beliefs. While past work has explored the outcomes associated with broad religious devotion or participation, it has not tested the impact of religious belief, let alone parsed belief into malevolent and benevolent components. Given the divergent effects of these two sides of religious belief cited in the literature above, and its important theoretical implications for understanding the origins and functions of the various facets of religions, we examine the impact of two widely recognized religious ideas: heaven and hell. Second, the present work presents one of the first direct experimental investigations of the consequences of such malevolent and benevolent concepts (Study 3), which, hitherto have been primarily examined with correlational designs [1], [2], [10].

## Study 1: Cross-national Comparisons

To measure the relationship between Heaven and Hell belief and subjective well-being at the cross-national level, we compared differences in subjective well-being between 63 countries against national rates of Heaven and Hell beliefs. In order to discount obvious alternative explanations, we controlled for macroeconomic (wealth, wealth inequality, unemployment and inflation), religious (belief in God and religious attendance), and social (civil liberties and socio-political stability) factors.

### Materials and Methods

We used national subjective well-being data from the 2005–2009 Gallup World Poll [8], a large, recent, high-quality survey of 455,104 respondents across 155 nations (minimum 1,000 per nation), conducted via telephone and face-to-face interviews. Responses produced two national variables of well-being: *life satisfaction rank* and *daily affect*.

#### Life Satisfaction Rank

National life satisfaction ranking was based on responses to questions probing overall life satisfaction (e.g. “*How happy are you with your life as a whole these days?*” – measured on a Cantril [15] scale ladder 0 (*worst possible life*) to 10 (*best possible life*) – for both present circumstances and what people expect in the five years time. From these future and present ratings, respondents were categorized as ‘thriving,’ (those who scored 7 or higher on present circumstances, and 8 or higher on future circumstances), ‘suffering’ (those who scored 4 or below on both categories), or ‘struggling’ (those in between the other two categories). The proportion of respondents in each category within a nation was used to determine an overall life satisfaction ranking for that nation. For example, Togo was ranked lowest, at number 155, with 1% of its respondents categorized as ‘thriving’ and 31% as ‘suffering.’ On the other hand, in Denmark, ranked at number 1, 82% of respondents were ‘thriving’ and only 1% ‘suffering’ (see Table 1 for descriptive statistics of key variables). National rankings were used because overall means were not yet publicly available at time of writing.

**Table 1:** Descriptive statistics for key variables in Study 1.

Measure/Item	Range	Mean	Standard Deviation
National Happiness Rank (lower is happier)	1 (Denmark) –155 (Togo)	77.10	44.73
Daily Experience (higher is happier)	5.0 (Togo) –8.4 (Panama)	7.04	0.85
Heaven Belief	16% (Vietnam) –100% (Various)	68%	0.26
Hell Belief	11% (Sweden, Germany) –100% (Various)	56%	0.28

#### Daily Affect

Daily affect was calculated using a different set of ten questions which asked respondents about their affect and experience during the prior day (example items: “Did you smile or laugh a lot yesterday?”, “Did you experience sadness during a lot of the day yesterday?” (reverse-scored), “Would you like to have more days just like yesterday?”). Respondents answered “Yes” or “No” and responses across this set of questions were combined to form a single overall score out of 10, where 0 would indicate that the respondent answered “No” for each of the ten questions, and 10 indicating that the respondent answered “Yes” for all of the questions. Then the respondents' scores from each country were averaged to form a national mean, which ranged from a low of 5.0 (in Togo) to a high of 8.4 (in Panama).

These two well-being variables – *life satisfaction rank* and *daily affect* – were only moderately correlated across nations, *r*(155)  = −.32, *p*>.001. Though one may expect a stronger correlation, it should be noted that these two variables capture different components of subjective well-being, as described by Diener and colleagues: the cognitive evaluation of one's life, and the affective happiness of one's day to day experiences, respectively [16]–[17]. Moreover, the two constructs have been shown to be predicted by different things; for example, having been a college graduate is related to life satisfaction, but has a minimal relationship with emotional well-being, whereas having headaches shows the opposite pattern [18]–[19]. We hypothesized that both, however, may be related to religious beliefs. Note that the Gallup World Poll, and other broad surveys of well-being like it, have been shown to be valid, reliable and cross-culturally comparable [18].

**Figure 1:**
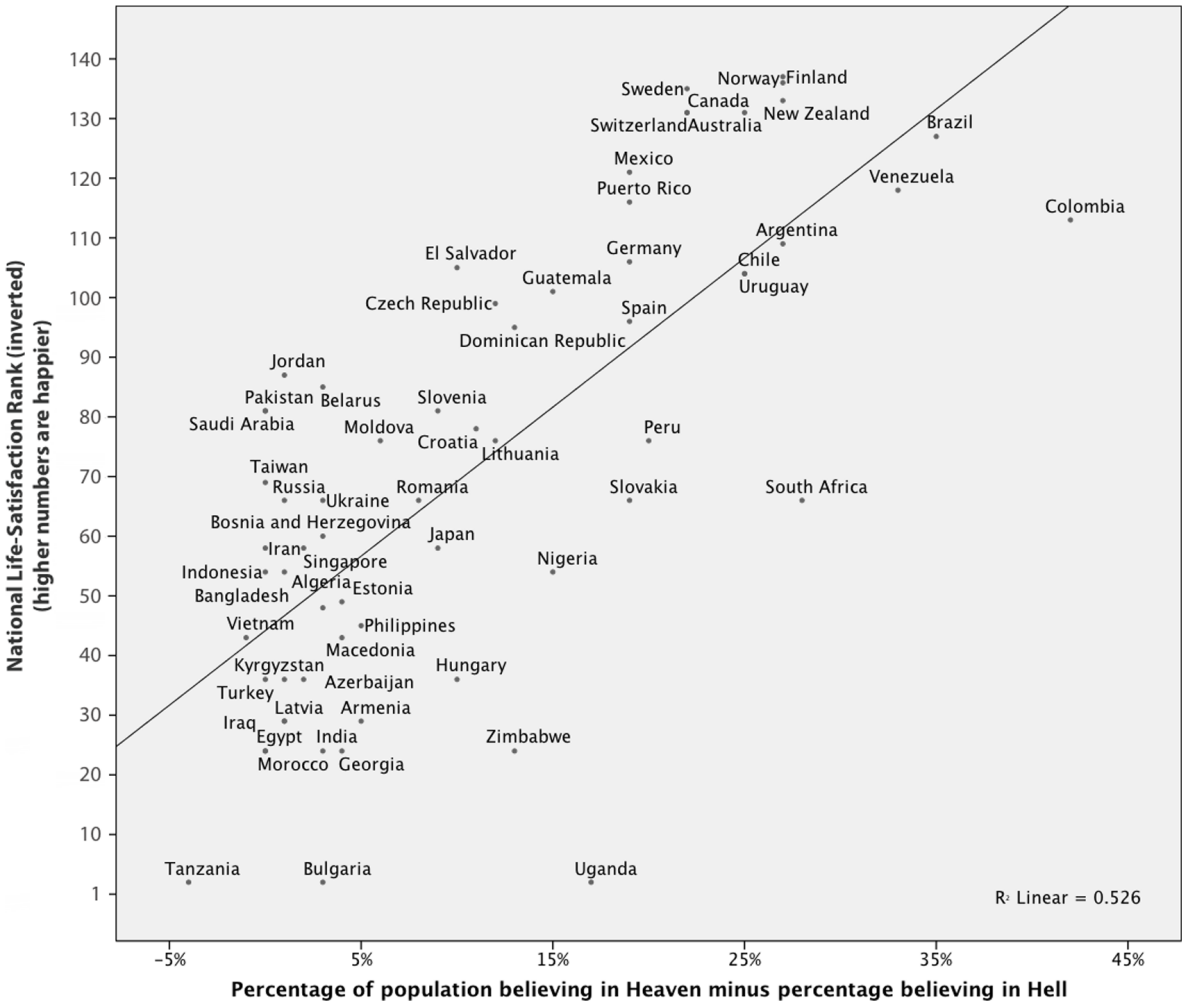
National Happiness Rank as a function of how much higher the proportion of a nation that believes in Heaven is compared to the proportion that believes in Hell. *Ranking is inverted such that nations higher up on the y-axis are happier. R^2^ = .53.*

**Figure 2:**
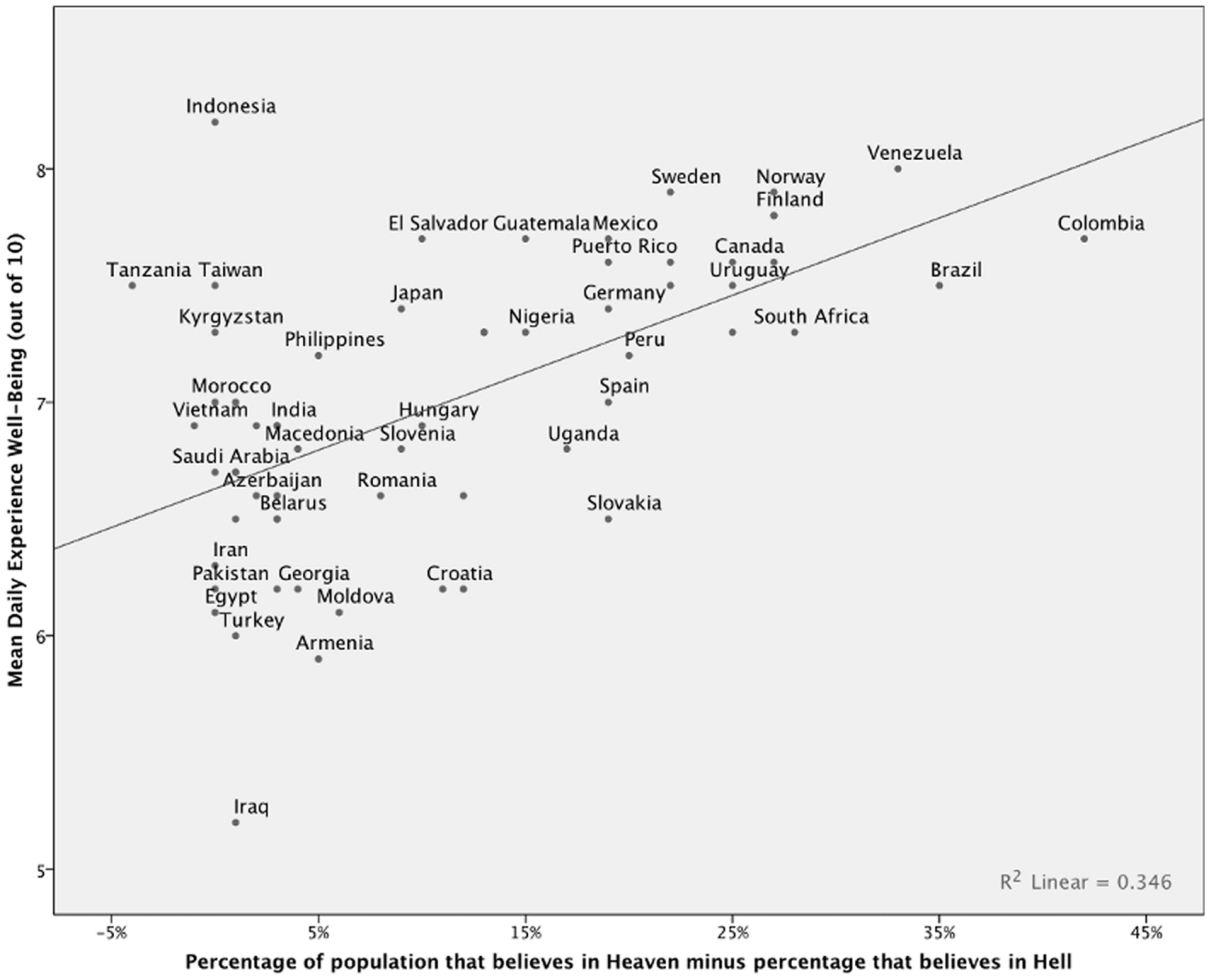
Daily Experiences Well-being as a function of how much higher the proportion of a nation that believes in Heaven is compared to the proportion that believes in Hell. R^2^ = .35.

Data on Heaven belief and Hell belief were extracted from fives waves of the World Values Survey (WVS) and European Values Survey (EVS) [8] collected between 1981 and 2007. In total, there were 146,562 participants from 63 countries (mean *n* per country  = 2326, range  = 387 (Dominican Republic) – 9569 (South Africa)). Values report the percentage of respondents endorsing belief in either Heaven (item f054) or Hell (f053). Belief in Heaven, Hell and God was assessed with the question, “Which, if any, of the following do you believe in?”, followed by a list of concepts including “Heaven,” “Hell,” and “God” Accepted answers were “Yes” and “No”. In order to succinctly visualize the relationship, Figures 1 and 2 use a difference measure created by subtracting the proportion of a nation's Hell believers from the nation's proportion of Heaven believers. Since nearly every nation has more people endorsing Heaven than Hell, this value is nearly always positive.

To discount alternative explanations, we included several covariates in our analyses which could be associated with various religious beliefs and well-being. Belief in God (f050) and religious attendance (f028) were drawn from the WVS and EVS. Religious attendance was assessed with the question, “Apart from weddings, funerals, and christenings, about how often do you attend religious services these days?”; response options were 1 =  More than once a week, 2 =  Once a week, 3 =  Once a month, 4 =  Only on special holy days/Christmas/Easter, 5 =  Other specific holy days, 6 =  Once a year, 7 =  Less than once a year, 8 = Never or practically never.

Gross Domestic Product per capita (logged), the Gini index of income inequality, and the inflation rate – all previously linked to well-being [20]–[22] – were taken from the 2011 CIA Factbook [23] (for nations where 2011 data were not available, the most recent data were used). Unemployment rates, also tied to well-being [24], were calculated as the average of the available data from 2006 to 2011, and pulled from the World Bank Databank [25]. Estimates of Political Stability and Absence of Violence and Terrorism from 2010 were drawn from the World Bank's Worldwide Governance Indicators [26]. All data are publicly available.

All variables were entered into a linear regression. Following recommendations by Simmons, Nelson & Simonsohn [27], results were calculated both with and without covariates (see Table 2). Listwise deletion was employed, thus there are 63 nations included in the beliefs-only analysis, and 52 included in the analysis with covariates.

**Table 2:** Predicting national happiness rank and daily experiences of well-being from heaven and hell beliefs in Study 1.

Predictor	Life Satisfaction Rank (inverted) (higher values indicate higher well-being)			Daily Experience Well-being		
	*F*	*R^2^*	*β*	*F*	*R^2^*	*β*
**Model 1: No covariates**	26.41***	.53		12.94***	.39	
Heaven Belief			1.64***			1.45***
Hell Belief			−1.86***			−1.51***
**Model 2: With covariates**	13.09***	.73		4.65***	.50	
Heaven Belief			1.74***			1.49**
Hell Belief			−1.51***			−1.38***
God Belief			−.23			−.22
Religious Attendance			−.01			.09
GDP per capita (log)			.44**			−.02
Gini Coefficient			−.07			.09
Inflation Rate			−.09			−.03
Unemployment Rate			−.09			−.27*
Stability & Absence of Violence			−.12			.19

denotes p<.05, ** denotes p<.01, *** denotes p<.001.

### Results

When controlling for each other and potential third variables, Heaven and Hell both emerged as significant, but divergent predictors of happiness. Notably, this is true regardless of whether happiness was assessed with the national life satisfaction ranking or the daily affect measure. Belief in Hell predicted lower life satisfaction ranking (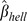 = −1.51, *p<*.001) and lower daily affect (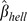 = −1.38, *p<*.001), whereas Belief in Heaven predicted higher life satisfaction ranking (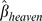 = 1.74, *p<*.001) and daily affect (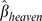 = 1.49, *p = *.001). These emerged as the strongest of all included predictors (see Model 1 in Table 2). Indeed, the two variables of specific religious beliefs – a belief in Heaven and Hell – alone predicted 53% of the cross-national variance as measured by life satisfaction rank and 35% of the cross-national variance in daily experiences well-being (see Figures 1 and 2). We note that the predictive ability of these measures remained when additional controls were entered in the model, suggesting that the relationship between beliefs in Heaven, Hell, and well-being is robust (see Model 2 in Table 2). Furthermore, the other measures of religiosity – belief in God and religious attendance – did not significantly predict well-being when questions about specific Heaven and Hell beliefs were included in our regression model. This underscores the importance of assessing the divergent benevolent and malevolent aspects of religion, which when combined may mask important differences.

These data indicate that beliefs in Heaven and Hell are strong and opposite predictors of well-being at the national level. However, while the cross-national comparison in Study 1 is illustrative, we note that it is based on a relatively small sample size of countries, which only allowed us to control for national level variables such as per capita wealth. Further, these limitations may have obscured differences that may result from religious variation. For instance, it is possible that Heaven and Hell beliefs are only related to well-being among adherents to Abrahamic religious tradition, which offers a somewhat consistent messages about the positive features of Heaven and the negative features of Hell. Given that only 8 of the 63 countries examined in Study 1 are countries in which a religion other than Islam or Christianity is the majority religion, we could not examine whether the observed relationships between Heaven and Hell beliefs and well-being are also present in non-Abrahamic countries with sufficient power.

As a result, in Study 2, we turned to individual-level data, which, though often noisier, provided a larger sample to investigate religious differences, and allowed us to control for individual variables such as sex, age and education level. We sought to test whether the pattern of cross-national results found in Study 1 is detectable at the individual level as well.

## Study 2: Large-scale correlational study

Using the WVS and EVS, we measured the association between life satisfaction and Heaven/Hell belief, again controlling for a number of associated variables.

### Materials and Methods

All variables were drawn from the same waves of the WVS and EVS as were used in the first study. Here, though, individuals' responses (*n* = 257, 597) were used, rather than aggregating data into a national average.

Because individuals living in the same country may respond to survey questions in a similar way, we used multi-level modeling to account for the possibility of within-country dependence. Heaven belief, Hell belief, God belief and religious attendance were the same as those used in Study 1. The dependent measure, subjective well-being, was assessed using a life satisfaction item (a170) asking “All things considered, how satisfied are you with your life as a whole these days? Using this card on which 1 means you are “completely dissatisfied” and 10 means you are “completely satisfied” where would you put your satisfaction with your life as a whole?” Belief in God (f050), Religious Attendance (f028), Age (×003), Age-squared, Sex (dummy coded, 1 = male, 2 = female; ×001), Education Level (×025), relative Income Level (1 = low, 2 = medium, 3-high; ×047r), Self-reported importance of friends (1 = Not at all important, to 4 = Very important, a002) and Self-reported importance of family (1 = Not at all important, to 4 = Very important, a001) were also included as covariates. The fitted model equation was
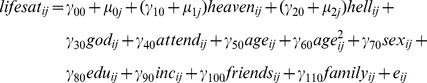
where 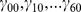 are fixed effects representing the mean intercept and regression coefficients at the individual (within-country) level, and 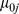, 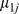 and 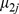, are a random intercept and random effects of heaven and hell. The variance of these random effects (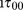,
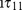
and 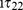) reveal the variability of the individual-level effects across countries. The software package lme4 in R was used to run the model [28]–[29].

### Results

Fixed effects analyses reveal the extent to which heaven beliefs and hell beliefs predict life satisfaction at the individual level (within countries), controlling for the effects of age, sex, relative income, religious attendance, and belief in god. Mirroring the pattern of results seen in Study 1, the belief in Heaven is associated with greater life satisfaction (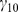 = .25, *p*<.001), but the belief in Hell is associated with less (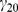 = −.28, *p*<.001) (See Table 3). While our focus was on the impact of heaven and hell beliefs on life satisfaction across countries, we note that random effects analyses did reveal that this relationship varied little for heaven beliefs (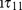 = .01) and a small to moderate amount for hell beliefs (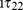 = .15).

**Table 3:** Predicting individual subjective well-being from heaven and hell beliefs in Study 2.

Variable	Coefficient	SE
Individual-level predictors		
Heaven Belief, *γ*_10_	.25***	(.07)
Hell Belief, *γ*_20_	−.28***	(.09)
God Belief, *γ*_30_	−.01	(.07)
Religious Attendance, *γ*_40_	.03***	(.01)
Age, *γ*_50_	−.04***	(.00)
Age-squared, *γ*_60_	.00***	(.00)
Sex, *γ*_70_	.21***	(.02)
Income, *γ*_80_	.54***	(.02)
Education Level, *γ*_90_	.03***	(.01)
Importance of Family, *γ*_100_	.31***	(.04)
Importance of Friends, *γ*_110_	.15***	(.02)
Intercept, *γ*_00_	6.12***	(.22)

*denotes p<.001.*

Although our primary interest was examining the relationship between Heaven beliefs, Hell beliefs, and well-being with the equation above, we also explored whether these relationships varied depending on a respondent's religious denomination. To do so, we categorized respondents by their reported religious affiliation into either (a) the Abrahamic tradition (e.g. Roman Catholic, Sunni Muslim; n = 180,843) or (b) the non-Abrahamic religion (e.g. Hinduism, Buddhism; n = 22,193). To test whether Heaven and Hell beliefs' relationship with well-being interacted with membership to these meta-religious groups, we conducted a new analysis with belief in hell (centered), belief in heaven (centered), whether the respondent adhered to an Abrahamic religion (dummy coded, −1 = no, 1 = yes), and the interaction terms (Hell belief X Abrahamic, Heaven belief X Abrahamic) entered into the fitted model, along with the same covariates from the main analysis above, all predicting well-being. The new model equation was:
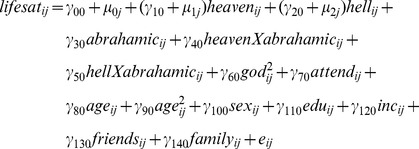



Analyses revealed that the observed relationship between Heaven beliefs and Hell beliefs did not vary by respondents' religious denomination. Indeed, the non-significant interaction terms indicate that the emotional correlates of heaven and hell beliefs are similar for Abrahamic and non-Abrahamic religious believers (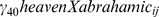 = .05, *p* = 0.33; 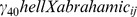 = −.06, *p* = 0.19), while the Heaven and Hell beliefs remained significant predictors of well-being. Thus, these results suggest that the divergent effect of Heaven and Hell beliefs on well-being does not differ between Abrahamic and non-Abrahamic adherents.

These findings complement the pattern seen in Study 1; Heaven and Hell beliefs have sizable, but divergent effects on well-being. The individual-level effects of belief in Heaven and Hell on happiness in this study are smaller here than the country-level effects in Study 1. However, the individual-level values are larger or comparable in size to other important predictors of life satisfaction, such as education level (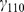 = .03, *p*<.001) and sex (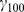 = .21, *p*<.001), though smaller than the effect of income (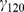 *= *0.54, *p*<.001).

Though we tried to discount obvious third variable explanations in Studies 1 and 2, both use correlational designs, which are limited in their ability to determine causation. While we suggest that a belief in Hell leads to lower levels of well-being, these data cannot rule out the possibility that individuals with low levels of well-being are more likely to adopt the belief in Hell or that some third variable is responsible for this pattern. Furthermore, even if the causal direction does run from Heaven and Hell beliefs to well-being, the correlational results leave open the possibility that these effects might be indirect – operating on intermediary phenomena – rather than direct. For example, Brañas-Garza and colleagues [10] have similarly used large datasets to show that belief in Heaven is more tightly related to religious practice and service attendance than is Hell. It is possible that the beliefs in benevolent and malevolent afterlives do not affect well-being directly, but do so via alternative pathways such as religious participation.

In order to clarify the specific causal relationships, we conducted Study 3, an experimental priming study in which we assigned participants to think about Heaven, Hell, or a control topic before reporting their current happiness. If Heaven and Hell beliefs have direct and divergent well-being consequences, we should observe happiness differences between participants in these two experimental conditions.

## Study 3: Experiment

### Notice of IRB Review and Approval-Amendment

Expedited Review as per Title 45 CFR Part 46.110, 63 FR 60366, # 7, 46.117(c)(2) ?The amendment submitted for the project identified above has been reviewed and approved by the University of Oregon Institutional Review Board (IRB) and Research Compliance Services using an expedited review procedure. This is a minimal risk study. This approval is based on the assumption that the materials, including changes/clarifications that you submitted to the IRB contain a complete and accurate description of all the ways in which human subjects are involved in your research.

### Methods

Four hundred and twenty-two American participants (*M*_age_  = 28.9, *SD* = 10.1, Range  = 18–71; 53.5% female (not all participants reported their sex and age)) completed a survey on Amazon's Mechanical Turk survey site in exchange for $0.35 each. Fifty-seven percent reported being religious believers, of which 82% were Christian (20.3% Catholic, 52.3% Protestant, 9.3% did not specify), 8% indicated Other, and Jewish, Muslim, Hindu and Buddhist participants made up the remaining 10%. Unlike the samples from Studies 1 and 2, who were randomly polled across the world, the participants in Study 3 were all self-described American residents who self-selected to participate in Mechanical Turk's set of online tasks for hire, and, in particular, the current study, which was advertised as “Autobiographical Memory and Mood”.

Participants were randomly assigned to one of three conditions. In the Hell condition, participants were asked to write 100–200 words about their conception of Hell, including its purpose and description. In the Heaven condition, participants were similarly asked to write about Heaven. In the control condition, which was designed to be neutral and non-religious, participants were asked to write about what they did yesterday.

Subsequently, participants were asked to rate the extent to which they were experiencing seven emotions – happiness, sadness, guilt, security, shame, fear and calmness – on a scale from 1 (“Very slightly or not at all”) to 5 (“Extremely”). Finally, participants completed a series of demographic questions, a suspicion probe, questions about their religious beliefs, and a manipulation check, in that order. The suspicion probe revealed that five participants (1% of sample) correctly guessed the hypothesis; these respondents were dropped from analyses, leaving a final sample of 417 participants (including these five participants did not significantly change the pattern of results). A manipulation check queried participants on the degree to which they thought about the primed topics (e.g. “Thinking back to your writing task, to what degree did you focus on the idea of Hell?”) on a scale from 1 (“Very slightly or not at all”) to 5 (“An extreme amount”). The check confirmed that participants in each of the three conditions thought more about the topic they were primed with, than the topics they were not (neutral condition: *t*(253)  = 5.82, *p*<.001; Hell condition: *t*(252)  = 4.87, *p*<.001; Heaven condition: *t*(254)  = 3.95, *p*<.001; The homogeneity of variance (HOV) assumption was violated in these manipulation check analyses (all Levene's test *p*<.05). Therefore, we present the Welch Test corrected values). Importantly, the manipulation check also confirmed that the degree to which participants reported thinking about their respective primed topic did not differ between conditions (*F*(2,412) <.25, *ns*). That is participants who wrote about Hell did not think about Hell more than participants who wrote about Heaven thought about Heaven. This suggests that all three primes were equally engaging and that effects cannot be attributed to artifacts of certain primes being more effective than others.

Following Studies 1 and 2, we predicted that participants assigned to think about Heaven would report higher levels of positive emotion and lower levels of negative emotion than those in the control condition. Similarly, we expected participants assigned to think about Hell to report lower levels of positive emotion and higher levels of negative emotion than those in the control condition.

### Results

Individual one-way Analysis of Variance (ANOVA) tests were conducted to examine the effect of the priming manipulations on Happiness (*F*(2, 412)  = 6.14, *p* = .002), Sadness (*F*(2, 388)  = 3.32, *p* = .037), an aggregated average of the three Positive Emotions minus the four Negative Emotions (*F*(2, 349)  = 4.95 *p* = .008).

Breaking these initial results down with planned contrasts revealed that the emotion differences were driven entirely by the Hell prime. Participants who wrote about Hell reported significantly less happiness and more sadness than those who wrote about Heaven (*t_happiness_*(407)  = 2.60, *p* = .010; *t_sadness_*(384)  = 2.29, *p* = .023), or those in the neutral writing condition (*t_happiness_*(407)  = 3.44, *p* = .001; *t_sadness_*(384)  = 2.32, *p* = .021) (see Table 4 for all means and SDs). Notably, and supporting Shariff & Rhemtulla [2] and others' suggestion about the Supernatural Punishment Hypothesis, those writing about Hell also reported more fear than those in the Heaven (*t_fear_*(361)  = 2.62, *p* = .009) and control conditions (*t_fear_*(361)  = 2.63, *p* = .009). In total, subtracting the average of all negative emotions from the average of all positive emotions, those who wrote about Hell reported more emotional negativity than those in the Heaven (*t_all_emo_*(344)  = 2.44, *p* = .015) and control conditions (*t_all_emo_*(344)  = 3.08, *p* = .002). Those writing in the Heaven and control conditions did not significantly differ on any of these measures (*t*s<1.0, *p*s>.35).

**Table 4:** Means and standard deviations for the experimental conditions in Study 3.

Measure	Condition	Mean	SD
Happiness	Control	3.35^a^	1.06^a^
	Heaven	3.25^a^	1.01^a^
	Hell	2.91^b^	1.03^a^
Sadness	Control	1.65^ a^	0.91^a^
	Heaven	1.65^ a^	0.84^a^
	Hell	1.92^ b^	1.00^a^
Fear	Control	1.46^ a^	0.81^a^
	Heaven	1.46^ a^	0.81^a^
	Hell	1.75^b^	0.96^a^
Positive Emotion	Control	3.50^ a^	0.91^a^
	Heaven	3.43^ a^	0.89^a^
	Hell	3.24^ b^	0.87^a^
Negative Emotion	Control	1.89^ a^	0.76^a^
	Heaven	1.97^ a^	0.94^a^
	Hell	2.29^ b^	1.05^b^
Positive *minus* Negative Emotion	Control	1.56^ a^	1.43^a^
	Heaven	1.44^ a^	1.58^a^
	Hell	0.93^b^	1.63^b^

Note: Means with different superscript values (i.e. ^a^ and ^b^) are significantly different from one another at the *p*<.05 level. Assumptions of homogeneity of variance were violated and corrected for when comparing the Control versus Hell condition for Negative Emotion.

What relationship does dispositional religious affiliation have with emotion ratings? Collapsed across condition, those who identified as religious believers reported higher levels of happiness (*M* = 3.40, *SD* = 1.07) than those identifying as religious non-believers (*M* = 3.04, *SD* = 1.01, *t*(407) = 3.46, *p* = .001), replicating a consistent finding regarding the self-reported mood benefits of religious identification [30]. However, there was no significant interaction between religious identification and condition (*F*(2,198)  = .19, *p* = .824); religious believers and non-believers both showed more emotional negativity when writing about Hell compared to the control condition (*t_believers_*(150)  = 2.35, *p* = .02; and *t_non-believers_*(190)  = 1.99, *p* = .049). It is notable that reflecting on Hell negatively affected well-being, regardless of whether the participant identified as a religious believer. There are numerous interpretations for this, and it is a ripe avenue for future investigation.

## Discussion

Three studies showed that heaven and hell beliefs are associated with markedly divergent well-being outcomes. Two large-scale correlational studies conducted with international data sets showed that, controlling for each other, Hell beliefs were associated with lower well-being at the national level and individual level, whereas Heaven beliefs were associated with higher well-being. Furthermore, an experiment using an online sample of Americans shows consistent findings; priming participants with Hell leads to lower levels of positive emotion and higher levels of negative emotion, compared to controls.

The results of Study 1 demonstrate that Heaven and Hell beliefs have divergent effects both on the day-to-day affective experiences of joy and sadness, as well as on overall evaluations of life satisfaction, suggesting that religious beliefs might relate to multiple levels of well-being. Similarly, Study 2 replicates the link between heaven and hell beliefs with well-being at the individual level. That said, while Studies 1 and 2 provide compelling evidence for such links, the correlational nature of our investigations preclude causal conclusions regarding the direct impact of either Heaven or Hell beliefs. However, the results of Study 3's suggest that the beliefs do have a causal impact on well-being. It remains possible that the well-being differences between the two types of beliefs seen in Studies 1 and 2 are the result of multiple pathways, Study 3's results support the conclusion that one of these is the direct impact of thinking about Hell. This interpretation should be taken with some caution, though, considering the entirely American and predominantly Christian and non-religious sample. Though Amazon's Mechanical Turk has been shown to be somewhat more representative than undergraduate samples [31], it can by no means be taken to be globally representative.

Nevertheless, our finding that certain religious beliefs are consistently related to lower levels of well-being adds nuance to the general finding that religion is tied to greater well-being [30]. Although we replicate this general finding in Study 3, where religious believers reported higher positive affect and lower negative affect than did non-believers, all aspects of religion do not seem to be created equal in this regard. In fact, in our experimental test, neither Hell nor Heaven belief contributed to an increase in mood above what was found in our control condition. Though the heaven writing task likely did not capture the whole spectrum of mood and security benefits that a long-standing belief in heaven may actually afford, the absence of an effect lends support to the possibility that the well-being benefits of religiosity derive from its social aspect, not its beliefs [7] (this hypothesis is further supported by the observation that in our cross-national analyses, after controlling for wealth, wealth inequality and political stability/absence of violence, the rate of religious attendance in a nation emerged as a significant predictor positive predictor of daily experienced well-being (*β* = .35, *p = *.040), but the rate of belief in God did not (*β* = .03, *p = *.864). Diener, Tay & Myers [9], for instance, showed that religiosity only relates to well-being in those areas with religious majorities.

### Why Hell?

If the belief in Hell has reliably negative effects on well-being, why has it persisted? In the introduction, we cited evidence for the association between Hell beliefs and ethical behavior. Thus, the belief in Hell, and religious malevolence more generally, may contribute to the encouragement of rule following, through the deterrence value of supernatural punishment, but may do so at the cost of well-being. This creates an intriguing trade-off between the interests of the group, which benefit from the ethical behavior of the group's members, and the interest of the individual, who shoulders the emotional costs of a society that follows norms out of fear. From a cultural evolutionary perspective, different societal circumstances could shift the balance of this tradeoff. For example, where rule-following is well organized by secular institutions, supernatural punishment may provide less added value on this front [32]. In these societies, one might expect religions to shift towards a more benevolent tone – especially in a competitive religious market where such a benevolent tone may be more attractive to potential converts than fire, brimstone and other aspects of supernatural malevolence. Future research could investigate this possibility by examining conversion rates among religious sects that differ on these dimensions.

In sum, the current findings join a growing literature examining the different psychological impact of different concepts often conflated together as ‘religion’ [2], [33], [34]. Though certain of these religious concepts may be associated with greater well-being, the belief in Hell appears not to be one of them.

## Supporting Information

Datafile S1 **Data for Study 3.**
(SAV)
